# Targeted Metabolic Profiling of Urine Highlights a Potential Biomarker Panel for the Diagnosis of Alzheimer’s Disease and Mild Cognitive Impairment: A Pilot Study

**DOI:** 10.3390/metabo10090357

**Published:** 2020-08-31

**Authors:** Ali Yilmaz, Zafer Ugur, Halil Bisgin, Sumeyya Akyol, Ray Bahado-Singh, George Wilson, Khaled Imam, Michael E. Maddens, Stewart F. Graham

**Affiliations:** 1 Beaumont Research Institute, Beaumont Health, 3811 W. 13 Mile Road, Royal Oak, MI 48073, USA; ali.yilmaz@beaumont.org (A.Y.); zaferugur34@gmail.com (Z.U.); Sumeyya.Akyol@beaumont.org (S.A.); ray.bahado-singh@beaumont.org (R.B.-S.); George.Wilson@Beaumont.edu (G.W.); Khaled.Imam@beaumont.edu (K.I.); Michael.Maddens@beaumont.org (M.E.M.); 2 William Beaumont School of Medicine, Oakland University, Rochester Hills, MI 48309, USA; 3 Department of Computer Science, Engineering and Physics, University of Michigan-Flint, Flint, MI 48073, USA; bisgin@umich.edu

**Keywords:** Alzheimer’s disease, mild cognitive impairment, metabolomics, urine, biomarkers, machine learning, J0101

## Abstract

The lack of sensitive and specific biomarkers for the early detection of mild cognitive impairment (MCI) and Alzheimer’s disease (AD) is a major hurdle to improving patient management. A targeted, quantitative metabolomics approach using both ^1^H NMR and mass spectrometry was employed to investigate the performance of urine metabolites as potential biomarkers for MCI and AD. Correlation-based feature selection (CFS) and least absolute shrinkage and selection operator (LASSO) methods were used to develop biomarker panels tested using support vector machine (SVM) and logistic regression models for diagnosis of each disease state. Metabolic changes were investigated to identify which biochemical pathways were perturbed as a direct result of MCI and AD in urine. Using SVM, we developed a model with 94% sensitivity, 78% specificity, and 78% AUC to distinguish healthy controls from AD sufferers. Using logistic regression, we developed a model with 85% sensitivity, 86% specificity, and an AUC of 82% for AD diagnosis as compared to cognitively healthy controls. Further, we identified 11 urinary metabolites that were significantly altered to include glucose, guanidinoacetate, urocanate, hippuric acid, cytosine, 2- and 3-hydroxyisovalerate, 2-ketoisovalerate, tryptophan, trimethylamine N oxide, and malonate in AD patients, which are also capable of diagnosing MCI, with a sensitivity value of 76%, specificity of 75%, and accuracy of 81% as compared to healthy controls. This pilot study suggests that urine metabolomics may be useful for developing a test capable of diagnosing and distinguishing MCI and AD from cognitively healthy controls.

## 1. Introduction

Alzheimer’s disease (AD) is the most common neurodegenerative disease and currently lacks robust, non-invasive, diagnostic biomarkers [1]. The prominent characteristics of AD are the accumulation of β-amyloid plaques and tau tangles, which cause neuronal damage or loss of function [2]; however, the actual biochemical basis for neurodegeneration is poorly understood [3]. According to the Alzheimer Association’s annual report (2019), approximately 5.8 million Americans currently suffer from AD and it is anticipated this number will rise to 13.8 million in 2050. AD is the 6th leading cause of death in the United States and it is believed to cost ~$290 billion per year in health care [4]. The etiopathogenesis of AD is thought to begin decades before symptoms become apparent, and once symptoms such as memory loss, language problems, and other cognitive problems arise it is too late to treat the disease as the damage has already occurred [5]. The identification of early diagnostic biomarkers capable of identifying those people with AD years before irreversible brain damage has occurred is the number one priority for most grant-awarding institutions.

Mild cognitive impairment (MCI) is believed to be the intermediate stage between normal cognition function and dementia [6]; however, in some instances, MCI patients return to a normal cognitive state even though they have an increased incidence of developing dementia [7]. The conversion rate of MCI to AD is roughly about 10% per year, which increases with time [8]. To determine if a MCI patient has any underlying AD pathology, patients can undergo a number of neuro imaging tests, including magnetic resonance imaging, 11C-Pittsburgh compound-B positron emission tomography (PIB-PET), fluorodeoxyglucose positron emission tomography (FDG-PET), and identification of potential CSF markers [7]. However, all of these tests are cost prohibitive and are not always available. Therefore, improving how we diagnose MCI and those at greatest risk of phenotypically converting to AD is critical for the development of crucial medical interventions to slow or stop the progression of the disease [9].

Metabolomics involves the comprehensive analysis of small-molecule metabolites in a given biological matrix and their response to disease, drugs, diet, and lifestyle [3,10]. Our group and others have demonstrated the potential of metabolomics to successfully differentiate neurodegenerative diseases from healthy controls, while revealing insights into the underlying biochemistry [3,11,12,13,14,15,16,17,18,19,20,21,22]. However, these studies have not shown to have any clinical utility.

Through using proton nuclear magnetic resolution (^1^HNMR) spectroscopy and high-performance liquid chromatography–tandem mass spectrometry (LC-MS), a great deal of progress has been made toward metabolically profiling urine. While urine may be considered a waste product, it is considered as a valuable diagnostic biofluid [23]. It is readily available, non-invasive to collect, and provides a direct readout of what is happening within the body, making it an ideal matrix to identify a potential biomarker panel for AD [24,25,26,27]. One of the many problems associated with metabolomics is that no single platform can measure the entire metabolome. Therefore, combining ^1^H NMR and LC-MS enhances our coverage of the urine metabolome, increasing the likelihood that we will identify clinically viable potential biomarkers of MCI and AD.

For the first time we present a targeted, quantitative metabolomics approach that combines targeted LC-MS and ^1^H NMR to biochemically profile urine from MCI and AD sufferers and compare them with cognitively healthy age- and gender-matched controls. Our overriding goal is to determine if we can identify urinary metabolites that can be used to diagnose those patients with MCI and AD.

## 2. Results

**Figure 1:**
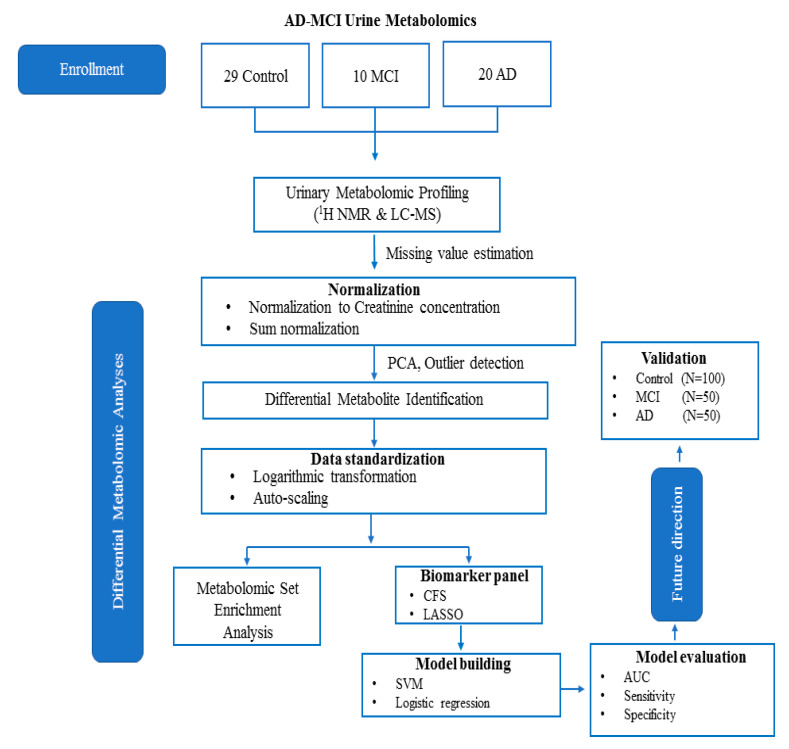
The current workflow and future direction of the current study as described herein.

The workflow for this study is shown in Figure 1. A total of 20 AD patients, 10 MCI sufferers, and 29 age- and gender-matched cognitively healthy controls (HC) were included in the study. We accurately identified and quantified a total of 142 metabolites using ^1^HNMR and 51 metabolites using DI-LC-MS/MS, respectively. Some degree of overlap was observed across the two platforms when measuring metabolite concentrations (n = 20). To account for this, we took the average of both measurements. In total, we accurately measured and quantified 173 metabolites in urine. Using Principal Component Analysis (PCA), we identified no class-specific subjects outside the 95th percentile (Supplementary Figure S1a–c). Using the metabolite concentrations, three pair-wise univariate and multivariate statistical comparisons were carried, including HC vs. MCI, MCI vs. AD, and HC vs. AD. Table 1 reports the results of multigroup comparisons of important demographic factors, such as age and gender. The results of the analysis of variance (ANOVA) revealed that neither gender nor age were statistically different between the groups (*p* < 0.05). Table 2 lists the results of the univariate analysis comparing the mean concentrations of urinary metabolites, showing significant changes between HC with MCI sufferers. As shown in the table, of the recorded metabolites, 17 metabolites were considered statistically significantly different (*p* < 0.05) between HC and MCI patients.

**Figure 2:**
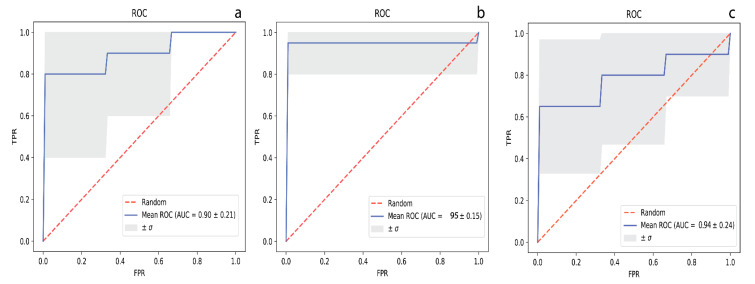
The respective receiver operating characteristic (ROC) curves with representative area under curve (AUC) values for distinguishing (**a**) HC from MCI, (**b**) MCI from AD, and (**c**) HC from AD, respectively.

Table S1 lists the performance values for the metabolite panel identified by CFS and LASSO that were deemed the most discriminative compounds when HC were compared to MCI sufferers. Using a 10-fold cross-validation method, we report each model’s evaluation as averages and standard deviations (n = 10 rounds). Figure 2a displays a ROC curve of the SVM model based on the metabolites, as highlighted by the CFS algorithm. The model was developed using the concentrations of isoleucine, acetate, trimethylamine n-oxide, kynurenine, C2, SDMA, malonate, and 5-aminopentanoate, and performed well with an AUC (95% CI) = 0.90 (0.874−1.000), with corresponding sensitivity and specificity values equal to 0.75 (0.923−1.000) and 0.77 (0.911−1.000), respectively.

The results of the univariate analysis for MCI vs. AD are available for metabolites whose levels show significant changes in Table 2. Of the recorded data, a total of 24 metabolites were found to be at significantly different concentrations in urine (*p* < 0.05) between MCI and AD sufferers. Using the concentrations of glucose, guanidinoacetate, urocanate, hippuric acid, cytosine, 2- and 3-hydroxyisovalerate, 2-ketoisovalerate, tryptophan, and malonate (Table S2), we developed a SVM model with an AUC (95% CI) = 0.95 (0.874−1.000) (Figure 2b), with corresponding sensitivity and specificity values of 0.78 (0.923–1.000) and 0.80 (0.911−1.000), respectively, following 10-fold cross validation.

Univariate analysis of the urinary metabolomics data revealed that of the 173 metabolites, only 9 of them were at statistically significantly different concentrations between cognitively healthy controls and AD sufferers (Table 2, *p* < 0.05). Among the diagnostic models tested, logistic regression performed the best. Using the concentrations of 2-hydroxyisovalerate, acetate, ethanolamine, pyridoxine, 2-hydroxybutyrate, and alpha-ketoisovalerate, we developed a diagnostic model with an AUC = 0.90 (0.821–1.000) (Figure 2c) and with sensitivity and specificity values of 0.88 and 0.78, respectively (Table S3).

## 3. Discussion

The accurate diagnosis of AD remains a clinical challenge in patient care, especially during the prodromal phase when treatment is most likely to be effective. In addition, another important challenge is to identify those people at greatest risk of phenotypically converting from MCI to AD. Current diagnostic approaches such as positron emission tomography (PET) and cerebrospinal fluid (CSF) biomarkers remain cost prohibitive and are not always available to specific patient populations across the globe. Therefore, we believe it is imperative to develop an inexpensive, widely available tool for the early diagnosis of AD. In this study, we target urine for those very reasons, as it is sterile, easy-to-obtain in large volumes, largely free from interfering proteins or lipids, and chemically complex.

Other researchers have also demonstrated the benefit of combining urine and metabolomics for the study of AD. For example, Yu et al. (2017) recently reported a global metabolomics study where the profiled urine samples were harvested from APPswe/PS1dE9 (APP/PS1) transgenic mice. In this study, they describe changes in metabolism and highlight potential biomarkers for the early diagnosis of AD [28]. Fukuhara et al. (2013) employed a NMR-based metabolomics approach to biochemically profile urine from tau amyloid precursor protein (TAPP) mice and found that the urine metabolome is perturbed in those mice considered to have AD, even before the hallmark symptoms of the disease become apparent [29]. A further advancement to urine metabolomics has been the incorporation of isotopically labelled standards, which have enabled a more detailed view of perturbations in metabolism [30]. As AD pathology and tissue loss progress, it has been proposed that the fragile double bonds in unsaturated fatty acids within the brain will increase, causing them to break down, and ultimately to be excreted in the urine. As such, Castor et al. (2020) recently reported that the levels of C7 to 10 increased in the urine of those patients with AD as compared with controls [31]. While we provide only a mere snapshot of urine metabolomics and AD, to the authors’ knowledge this is the first study to employ quantitative and global metabolomics approaches to profile urine obtained from patients with AD and individuals suffering from MCI and to compare them with the age- and gender-matched cognitively healthy controls. Although some degree of overlap exists between platforms (20 metabolites), each analytical methodology identifies a markedly unique class of metabolites. As metabolomics is highly reliant on a variety of sensitive analytical tools and due to the differences in the physiochemical properties of metabolites, there is currently no single analytical platform that is capable of detecting all metabolites in any biological matrix. Therefore, we combined 1D ^1^H NMR spectroscopy and targeted MS-based metabolomics to generate a more comprehensive metabolic profile, which yields superior diagnostic models. Moreover, given the complexity and heterogeneity of AD, combining metabolomics data obtained from multiple platforms may better reflect the etiology and provide new insights into the underlying biological processes behind the disease [32].

To account for any potential technical differences in sample preparation and data acquisition prior to employing any supervised classification approaches, we employed PCA to each individual group to ensure that systematic variation did not overshadow the biological variation. Our results showed that variation due to sample collection was negligible, as evidenced by the scores plots in Figure S1a–c. Having confirmed that there were significant differences in metabolite concentrations when those three groups were compared in a pair-wise manner, we aimed to investigate the changes in the urine metabolome as a direct consequence of AD (or MCI) and to systematically evaluate the accuracy of diagnosis using various artificial intelligence (AI) platforms in order to include SVM and logistic regression. We used variable importance functions such as LASSO and CFS to rank the features according to the contribution of each metabolite to classification performance. We performed a 10-fold CV to optimize the SVM parameters where accuracy or true diagnosis ratio was the single criterion. Our parameter space was logarithmically designed and exhaustively visited to seek the best accuracy. This is common practice during the optimization process of SVM models.

When comparing cognitively healthy controls vs. MCI, we found that (regardless of the variable selection algorithm used) SVM models performed the best for diagnosing MCI with respect to AUC, sensitivity, and specificity values (Table S1). In particular, our SVM model performed very well, with an impressive AUC (0.95) and encouraging sensitivity (0.75) and specificity (0.77) values. These reported values are comparable to those obtained by Mapstone et al. (2014), who used plasma metabolomics to differentiate MCI from controls [33].

Notably, we found that regression and linear SVM performed equally well. The SVM algorithm searches an optimal hyperplane separating the samples from two groups with a maximum distance to the training observations, which is called the margin. Simply put, when classes are overlapped, SVM is constructed by minimizing the cost of the training points that are on the wrong side of the classification boundary. SVM can also be extended to nonlinear boundaries by utilizing kernel functions to map the training observations to a higher dimensional space [34]. Contrastingly, logistic regression models predict the probability of a sample being a member of either group for a set of metabolite intensities. The probabilities are modeled as a function of intensity and the model coefficients are estimated by maximizing the log likelihood function [35].

The same procedure was applied with the top thirteen and five metabolites identified using the CFS and LASSO methods for distinguishing MCI from AD, respectively (Table S2). We used the concentration values of these metabolites to develop SVM and logistic regression models, which accurately distinguish MCI from AD sufferers. Of those models, the SVM model achieved the best diagnostic performance, with AUC = 0.95, sensitivity = 0.78, and specificity = 0.80. AD differed from MCI samples due to increases in PC ae C36:4, SM C26:0, PC ae C36:0, and decreases in acetic acid and acetone. The data suggest supportive energy pathways that connect proteins to glucose metabolism are affected. The dysregulated levels of tryptophan, alanine, and isoleucine may be associated with the defective pyruvate and acetyl CoA conversion of these amino acids, promoting the citric acid cycle to gain energy, thus altering how the brain is fueled during AD pathogenesis [36]. In contrast, altered levels of 2-hydroxybutyric acid and glucose in urine may be useful as early indicators of insulin resistance in non-diabetic AD and MCI patients. Moreover, elevated urine 2-hydroxybutyric acid levels predict worsening glucose tolerance in these patients. Another metabolite associated with energy metabolism found to be at significantly decreased concentrations in the urine of AD sufferers was guanidinoacetate. It is one of the intermediate metabolites that is directly involved in creatine synthesis in the brain and skeletal tissues. As with creatine, it is partly phosphorylated within the brain and muscle cells, as demonstrated by phosphorous magnetic resonance spectroscopy (^31^P-MRS) studies, while serving as an alternative source of high-energy phosphates in the skeletal muscle of guanidinoacetate methyltransferase-deficient mice, providing a certain degree of compensation for energy buffering and transport [37].

Vitamin B6 (pyridoxine) was the only water-soluble vitamin to be significantly increased in the urine of AD sufferers when compared with controls. B6 has plays a role in a diverse range of biochemical reactions that help regulate basic cellular metabolism, including amino acid, carbohydrate, and lipid synthesis, therefore influencing overall physiology [38]. Although controversial, vitamin B6 has been implicated as one of the protective factors against cognitive decline and AD [39]. Consumption of the vitamins pyridoxine, folate, and cobalamin at dosages of 20, 0.8, and 0.5 mg per day, respectively, for at least one year has been suggested to decrease brain atrophy and plasma total homocysteine levels, which have direct neurotoxic effects and are linked to brain atrophy in AD [40].

Although several variable selection algorithms have been routinely used in the field of metabolomics, such as in LASSO [41], CFS [42], and COR-LVQ [43]. It is unclear which, if any, of those methods are the most appropriate for analysis of a metabolomics dataset. In this regard, we attempted to systematically evaluate the performance of two machine learning approaches (SVM and logistic regression) through classification of the accuracy rate, sensitivity, and specificity when utilizing a panel of metabolites provided by CFS and LASSO, respectively. Interestingly, models built using a panel of urinary metabolites selected by the CFS method provided better AUC and sensitivity, however models utilizing a panel of metabolites identified by LASSO as being important were more specific to AD and MCI. Moreover, we found LASSO to be the most conservative selection algorithm, as in almost all cases it provided a subgroup of metabolites as the most significant for diagnosis.

Finally, a notable observation of this targeted metabolomics approach is that both variable selection algorithms selected metabolites measured by ^1^HNMR, making it the technique of choice when analyzing urine for potential biomarkers of MCI and AD.

## 4. Materials and Methods

### 4.1. Urine Samples

Human urine samples were collected from adult volunteers (20 AD, 10 MCI, and 29 Control patients). The diagnosis and evaluation of patients with AD and MCI sufferers by a geriatrician and neurologist or geriatric psychiatrist was made according to the criteria of the National Institute of Neurological and Communicative Disorders and the Stroke (NINCDS) and Alzheimer’s Disease and Related Disorders Association (ADRDA) [44]. Participants underwent a focused history and physical examination (assessing motor strength and tone, the existence of a tremor, sensation, balance (Romberg), and gait) to include an exhaustive cognitive testing battery routinely utilized in the Geriatric Clinic at Beaumont health, including: MMSE, SLUMS, CLOX-I, CLOX-II, trailmaking A, and trailmaking B, and geriatric depression scale testing (Table S4). The study was approved by the Ethics Committee of the William Beaumont Research Institutional Review Board (IRB# 2014-038). In metabolomics, to obtain accurate, reproducible, and reliable metabolome data, optimized standard protocols are crucial for metabolome sample preparation [45]. The methods were carried out in accordance with the approved guidelines. Following the standard sterile procedures, midstream urine samples were collected from all the fasting volunteers in the morning in a polypropylene container. Each sample was kept no more than 2 h at 2–8 °C before further processing [46]. Urine samples were centrifuged at 16,100× *g* and 4 °C for 30 min, and supernatants that were then aliquoted into an Eppendorf tube (0.5 mL for each tube) were immediately frozen and stored at −80 °C for targeted metabolomics analysis. The pH of each sample was measured before processing and analysis. The average pH was 7.22 ± 0.64, which is within the normal range.

### 4.2. ^1^H NMR Analysis

#### 4.2.1. Sample Preparation and Acquisition

After thawing on ice, a 500 μL aliquot of urine was removed and placed in a 1.5 mL Eppendorf tube. In order to further remove the proteins, the samples were centrifuged at 12,000× g for 10 min at 4 °C, then 300 μL of the supernatant was transferred to a clean 1.5 mL Eppendorf tube. Subsequently, 35 μL of D_2_O and 15 μL of a standard buffer solution (11.667 mM disodium-2,2-dimethyl-2-silapentane-5-sulphonate (DSS), 730 mM imidazole, and 0.47% NaN_3_ in H_2_O) were added to the urine supernatant. The urine samples (350 μL) were then transferred to a standard 3 mm thin-walled glass NMR tube for ^1^HNMR spectral analysis. All ^1^HNMR spectra were randomly collected on a on a Bruker Ascend HD 600 MHz spectrometer equipped with a 5 mm TCI cryoprobe. All 1D ^1^HNMR spectra were acquired at 25 °C using the modified version of the first transient of the Bruker NOESY presaturation pulse sequence, providing a high degree of quantitative accuracy [47]. Spectra were collected with 128 transients and 16 steady-state scans using a 5 s acquisition time and a 5.1 s recycle delay.

#### 4.2.2. Metabolite Identification and Quantification

Prior to spectral analysis, all FIDs were zero-filled to 128K data points and line broadened by 0.5 Hz. The methyl singlet produced by a known quantity of DSS (1000 μM) was used as an internal standard for chemical shift referencing (set to 0 ppm) and for quantification. All ^1^H NMR spectra were processed and analyzed using a Chenomx NMR Profiler (v. 8.1) and normalized to creatinine.

### 4.3. DI/LC-MS/MS Analysis

Direct flow injection MS using the commercially available AbsoluteIDQ p180 kit (Biocrates Life Sciences AG, Innsbruck, Austria) was used for MS analysis of urine. This kit was analyzed on a Waters TQ-S mass spectrometer coupled to an Acquity I-Class ultra-performance liquid chromatography (UPLC) system. Samples were prepared according to manufacturer’s instructions. A standard flow injection protocol consisting of two 20 mL injections (one for the positive and one for the negative ion detection mode) was applied for all measurements. Multiple reaction monitoring detection was used for quantification. MetIDQ software (Biocrates Life Sciences AG, Austria) was used to control the assay workflow, including for sample registration and calculation of metabolite concentrations. Prior to further statistical analysis, all MS-acquired data were normalized to creatinine.

### 4.4. Statistical Analysis

To account for any dilution effects, the combined ^1^HNMR and MS data were sum normalized. A metabolite was conservatively excluded if it had >50% missing data. For all other metabolites, missing measurements were imputed with the median value for said compound. Interestingly, concentration values ranged over several orders of magnitude both inter- and intra-sample. Therefore, prior to multivariate analysis, we addressed this by log-transforming and autoscaling the data. Principal component analysis (PCA) was performed on the preprocessed data to identify any potential outliers. Using MetaboAnalyst (v.4.1) [48], a Student’s t-test was performed to determine if there were any significantly different metabolites between AD, MCI, and age-matched controls (*p* < 0.05) when compared pairwise. Non-normally distributed data were analyzed using a Mann−Whitney U test and a Bonferroni correction was applied to account for multiple comparisons. To determine if sample demographics were statistically significantly different, a one-way analysis of variance analysis (ANOVA) was conducted using the IBM SPSS Statistics toolbox (v. 24.0). To develop the predictive models based on the most informative metabolites, a variety of tools offering different statistical approaches were employed on log-transformed and auto-scaled metabolomics data. Feature selection algorithms such as least absolute shrinkage and selection operator (LASSO) [41] and correlation-based feature selection (CSF) [42] were applied using MetaboAnalyst [49] and the WEKA tool [50], respectively. During the variable selection step using the LASSO method, a stepwise variable selection method was utilized to optimize all of the model components. Logistic regression model building was carried out using the R statistical package. A 10-fold cross-validation (CV) process was employed to ensure the models were not overfitted and to assess the predictive power on an independent sample. We used Scikit-learn [19], a machine learning library in Python, to perform an exhaustive search to obtain the best C-γ pair on a grid that was laid out on exponentially varying C and γ values, i.e., C ∈ [10^1^, to 10^5^] and γ ∈ [10^−1^, to 10^−6^]. More specifically, we employed a 10-fold cross-validation process for all C-γ combinations, aiming to achieve the highest accuracy, which is the ratio of truly predicted samples. The area under the curve (AUC at 95% confidence interval), sensitivity, and specificity values were calculated to estimate the performance of both the logistic regression and SVM models.

Importantly, urine is one the most frequently studied biofluids in metabolomics, as it is non-invasive, easy to obtain in large volumes, is free of other confounding macromolecules (proteins or lipids), while its chemical complexity makes it particularly suitable for metabolomic investigations. However, one of the pitfalls of being highly complex is that it is difficult to detect the whole metabolome using a single analytical platform. This requires the use of complimentary platforms such as NMR and MS to increase coverage. Among the measured metabolites, NMR has been reported to detect approximately 400 small molecules in urine across various studies. In our study we confidently identified and quantified 150 metabolites. In contrast, using the Biocrates p180 kit as employed herein only enabled us to report 51 metabolites. This further emphasizes the need for multiomics platforms to study any given biomatrix. Additionally, the use of urine to diagnose AD is still in its infancy, and herein we report a proof of concept study piloting it as a potential biomatrix for said use. While we report encouraging results, we do still acknowledge the study’s limitations. Firstly, our sample number was relatively small, which could limit the statistical significance; however, it does demonstrate the potential for using this tool in conjunction with other measures for the accurate diagnosis of this complex disease. Further, our study was limited by the amount of clinical and demographic information available. However, we do feel our study does warrant further investigation using larger, more well-defined cohorts for the validation of our initial biomarkers of disease. If successful it could be a stepping stone to the development of a robust, objective test with clinical utility.

## 5. Conclusions

In the current study, for the first time we have combined data acquired using ^1^HNMR and DI-LS-MS/MS with several robust AI approaches to identify urinary biomarkers for the detection of AD and MCI. The predictive accuracies achieved during the course of the study have shown that indeed urine should be considered as a biomatrix to be utilized for early prediction of MCI and AD. In addition, we provide novel and biologically plausible insights into the metabolic basis of AD using urine metabolomics. This pilot study suggests that urine metabolomics may be useful for diagnosing MCI and AD sufferers. However, as mentioned, this study is limited by its small sample size and the lack of an independent validation cohort. Our aim is to validate our findings using a much larger independent cohort, for which our sample collections are ongoing.

## Figures and Tables

**Table 1:** Multigroup comparison of the available demographic information and corresponding *p*-values calculated using one-way ANOVA and Chi-square test.

	Controls	MCI	AD	*P*-Value
**n**	29	10	20	
**Age, Mean (SD)**	79.12 (6.28)	76.57 (9.37)	79.92 (9.11)	0.43 ^a^
**Gender**				
Male	13	5	9	0.56 ^b^
Female	16	5	11	

^a^ One way ANOVA, ^b^ Chi-square Test.

**Table 2:** Multigroup presentation of metabolite concentrations (uM). Those values highlighted in bold represent those metabolites considered to be at statistically significantly different concentrations (*p* < 0.05). Statistical significance for those metabolites considered to be normally distributed were calculated using a conventional Student’s t test. The significance of those metabolites found to be non-parametrically distributed (W) was calculated using a Wilcoxon signed-rank test.

Name	Mean (SD) of HC	Mean (SD) of MCI	Mean (SD) of AD	*P*-Value HC vs MCI	*P*-Value MCI vs AD	*P*-Value HC vs AD
**2-Hydroxybutyric acid**	2.774 (1.379)	2.431 (1.244)	4.271 (2.599)	0.4368 (W)	**0.01762 (W)**	**0.0423 (W)**
**2-Hydroxyisovaleric acid**	0.948 (0.352)	0.891 (0.351)	0.013 (0.014)	0.2522	**0.03331 (W)**	**0.0461 (W)**
**3-Hydroxybutyric acid**	3.938 (5.098)	2.559 (2.904)	3.295 (2.450)	**0.0495 (W)**	0.0795 (W)	0.0832(W)
**3-Hydroxyisovaleric acid**	3.861 (1.909)	2.833 (0.929)	3.393 (1.350)	**0.0048**	**0.0347 (W)**	0.1528 (W)
**5-Aminopentanoic acid**	3.955 (4.715)	2.882 (2.831)	3.445 (3.628)	**0.0308 (W)**	**0.0257 (W)**	0.7668 (W)
**Alpha-ketoisovaleric acid**	3.402 (1.864)	2.564 (1.548)	1.015 (0.904)	**0.0463 (W)**	**0.0411 (W)**	**0.0307 (W)**
**C6:1**	0.009 (0.007)	0.014 (0.012)	0.008 (0.008)	0.1643 (W)	**0.0166 (W)**	0.2476 (W)
**Cytosine**	6.279 (7.311)	11.623 (15.351)	19.403 (8.093)	**0.0487 (W)**	**0.0386 (W)**	0.06467 (W)
**D-Glucose**	14.035 (8.563)	9.128 (3.037)	13.541 (6.780)	**0.0336 (W)**	**0.01232**	**0.0204**
**Dimethylsulfone**	9.238 (7.167)	5.028 (3.839)	4.173 (5.835)	0.9646	**0.0190 (W)**	0.0820 (W)
**Guanidoacetic acid**	15.031 (9.884)	9.077 (3.838)	16.389 (7.515)	**0.0103**	**0.0038 (W)**	0.4371 (W)
**Hippuric acid**	55.489 (7.874)	40.655 (6.302)	57.376 (6.537)	0.3908 (W)	**0.0111 (W)**	0.9945
**Mannitol**	13.260 (4.916)	17.071 (6.889)	7.808 (6.440)	0.4368 (W)	**0.0429 (W)**	0.1414 (W)
**Methanol**	52.958 (6.169)	59.581 (5.870)	47.690 (3.050)	**0.0266 (W)**	**0.0021**	0.0552 (W)
**PC aa C32:0**	0.019 (0.430)	0.02 (0.001)	0.02 (0.003)	0.1850 (W)	**0.0403 (W)**	0.4136 (W)
**Trimethylamine**	0.958 (2.582)	3.073 (5.811)	1.197 (3.040)	**0.0121 (W)**	**0.0412 (W)**	**0.0439 (W)**
**Tryptophan**	22.649 (22.057)	20.443 (11.526)	17.337 (9.148)	0.4646	**0.0114 (W)**	0.8012 (W)
**Alanine**	7.553 (7.690)	6.386 (3.828)	7.401 (3.007)	0.8868 (W)	**0.0439 (W)**	0.7395 (W)
**Proline**	4.727 (2.369)	5.641 (3.053)	6.804 (3.828)	0.4954 (W)	0.3735 (W)	**0.0394**
**Pyridoxine**	0.976 (1.215)	0.477 (0.375)	0.390 (0.373)	0.2720 (W)	0.5884 (W)	**0.0249 (W)**
**Isoleucine**	1.563 (0.917)	1.283 (0.740)	0.968 (0.416)	0.7158 (W	**0.02364**	0.9438 (W)
**Myo-inositol**	18.945 (6.379)	15.869 (8.629)	16.034 (5.995)	**0.0331 (W)**	**0.0134**	0.3440 (W)
**Trimethylamine n-oxide**	10.229 (7.735)	19.907 (10.822)	18.864 (11.571)	**0.0425**	0.7488	**0.0134**
**Glycolic acid**	12.043 (7.354)	15.671 (9.141)	8.274 (4.972)	0.9370 (W)	0.3735 (W)	**0.0518**
**Acetic acid**	6.136 (1.867)	14.663 (2.450)	9.336 (2.758)	**0.0485 (W)**	**0.0103**	0.7548 (W)
**Acetone**	0.884 (0.802)	1.442 (1.767)	1.068 (0.907)	0.7856 (W)	**0.0446**	1.0000 (W)
**PC ae C36:4**	0.002 (0.001)	0.002 (0.003)	0.019 (0.034)	**0.0134 (W)**	**0.0495 (W)**	0.2720 (W)
**SM C26:0**	0.674 (0.974)	0.350 (0.876)	0.674 (0.974)	**0.0475 (W)**	**0.0457 (W)**	0.1643 (W)
**PC ae C36:0**	2.376 (0.769)	1.622 (3.323)	2.878 (1.428)	**0.02241**	**0.0403 (W)**	0.3934 (W)
**Caffeine**	2.934 (1.724)	1.962 (2.014)	2.274 (1.375)	**0.0491 (W)**	0.3115 (W)	0.0691 (W)
**Isobutyric acid**	1.237 (0.840)	1.698 (1.201)	2.776 (1.724)	**0.0406 (W)**	0.0646 (W)	0.0628 (W)

## Data Availability

The metabolomics and metadata reported in this paper are available at MetaboLights Archive (https://www.ebi.ac.uk/metabolights/mysubmissions?status=PRIVATE) via the MetaboLights partner repository with the dataset no. MTBLS1695. Username: ali.yilmaz@beaumont.org; study ID is MTBLS1695.

## Supplementary Materials

The following are available online at https://www.mdpi.com/2218-1989/10/9/357/s1.
