# Supplementary material for: Targeted Metabolic Profiling of Urine Highlights a Potential Biomarker Panel for the Diagnosis of Alzheimer’s Disease and Mild Cognitive Impairment: A Pilot Study
Source: Metabolites. 2020 Aug 31;10(9):357. doi: 10.3390/metabo10090357 (PMC7569858; doi:10.3390/metabo10090357)
Supplement: Supplementary file 1 [file metabolites-10-00357-s001.pdf]

metabolites

MDPI

Supplementary Materials

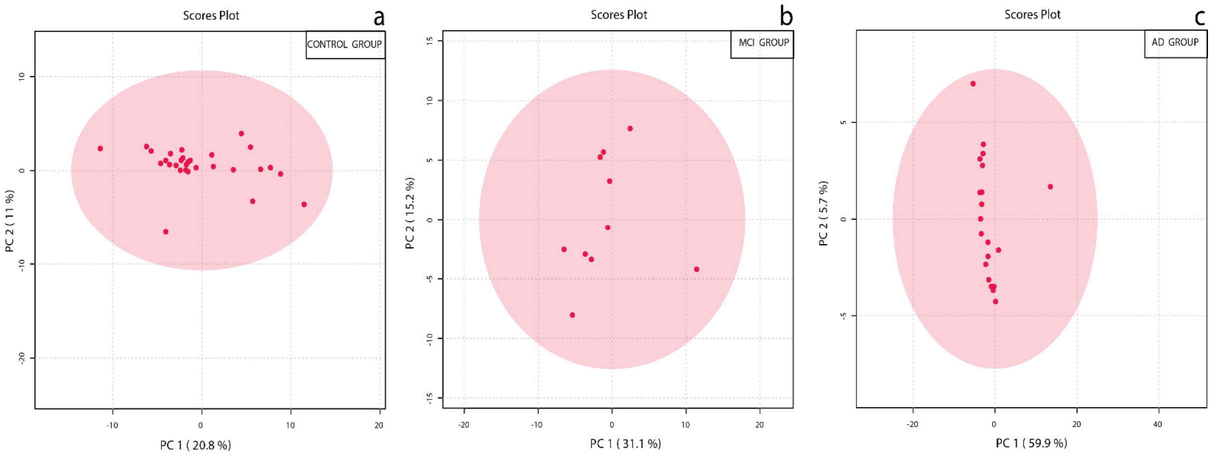

Supplementary Figure S1. Principal Component Analysis of the metabolomics data was used to test if there are any outliers in our cohorts due to systematic variation.

Supplementary Table S1. Panel of metabolites selected by the CSF and LASSO mathematical algorithms and their corresponding performance metrics when comparing healthy controls from MCI sufferers.

Metabolite Panel by CSF			Metabolite Panel by LASSO		
C14:1:OH			Isoleucine		
1-Methylnicotinamide			Acetate		
Acetate			Trimethylamine N-oxide		
Glucose			Kynurenine		
Isobutyrate			C2		
Trimethylamine N oxide			SDMA		
1-Methylhistidine			Malonate		
Hypoxanthine			5-Aminopentanoate		
L-Alanine					
Isoleucine					
3-Methylhistidine					
Methanol					
5-Aminopentanoate					
	**SVM**	**Logistic Regression**		**SVM**	**Logistic Regression**
**AUC**	95%	80%	**AUC**	90%	75%
**Sensitivity**	75%	77%	**Sensitivity**	77%	80%
**Specificity**	76%	67%	**Specificity**	75%	82%
**Accuracy**	82%	63%	**Accuracy**	81%	65%

Supplementary Table S2. Panel of metabolites selected by the CSF and LASSO mathematical algorithms and their corresponding performance metrics when comparing MCI from AD sufferers.

Metabolite Panel by CFS			Metabolite Panel by LASSO		
Glucose<br>2-Hydroxyisovalerate<br>3-Hydroxyisovalerate<br>Cytosine<br>Urocanate<br>2-ketoisovalerate<br>Glycolate<br>Guanidoacetate<br>Malonate<br>Hippurate<br>Tryptophan			Ethanol<br>Hippuric acid<br>Cytosine<br>Glucose<br>Guanidoacetate		
	**SVM**	**Logistic Regression**		**SVM**	**Logistic Regression**
**AUC**	95%	94%	**AUC**	81%	74%
**Sensitivity**	78%	80%	**Sensitivity**	75%	75%
**Specificity**	80%	64%	**Specificity**	79%	80%
**Accuracy**	77%	83%	**Accuracy**	81%	65%

Supplementary Table S3. Panel of metabolites selected by the CSF and LASSO mathematical algorithms and their corresponding performance metrics when comparing healthy controls from AD sufferers.

Metabolite Panel by CSF			Metabolite Panel by LASSO		
2-hydroxyisovalerate<br>Acetate<br>Ethanolamine<br>Pyridoxine<br>2-Hydroxybutyrate<br>Alpha-ketoisovalerate			2-hydroxybutyric acid<br>Dimethylamine<br>Proline<br>Glycolate<br>Isoleucine<br>Spermine<br>2-aminoadipate<br>Ethanolamine		
	**SVM**	**Logistic Regression**		**SVM**	**Logistic Regression**
**AUC**	94%	90%	**AUC**	79%	82%
**Sensitivity**	78%	88%	**Sensitivity**	78%	85%
**Specificity**	78%	78%	**Specificity**	78%	86%
**Accuracy**	80%	80%	**Accuracy**	88%	90%

Supplementary Table S4. The clinical parameters as collected for each individual recruited to our study. AD-Alzheimer's disease, MCI-Mild cognitive impairment; MMSE-Mini-menta state examination; CLOX-clock drawing task.

Study ID#	Group	MMSE Scores	Trails A/B	CLOX 1/2
SI 008	AD	29/30	NA	NA
MS 010	AD	0/30	NA	NA
MH 012	AD	17/30	NA	NA
AS 013	AD	29/30	NA	NA
NS 015	AD	20/30	116/364 sec	12/14
CM 022	AD	04/30.	NA	NA
BE 025	AD	29/30	Trails B 190	10/12.
FE 028	AD	9/30.	NA	NA
AD 032	AD	23/30	NA	9/11.
MB 044	AD	17/30	184/320	5/10.
RL 043	AD	29/30	74/198	6/11.
RM 045	AD	25/30	36/142	6/13.
JH 046	AD	28/30	52/140	6/11.
LB 051	AD	29/30	90/204	6/13.
VB 057	AD	27/30	194/25	10/9.
GE 059	AD	22/30	94/unable	9/11.
RW 064	AD	20/30	189/159	8/11.
JW 065	AD	12/30.	Unable	4/8.
AC 079	AD	16/30	NA	NA
CS 081	AD	20/30	277/unable	4/10.
JoJ 004	MCI	30/30	60/150 sec	
DC 016	MCI	30/30	Trails B 118	NA
JD 020	MCI	27/30	36/94	7/12.
AK 021	MCI	28/30	NA	NA
GS 052	MCI	29/30	90/204	6/13.
SP 066	MCI	29/30	54/108	14/13
CC 067	MCI	29/30	63/120	11/11.
RF 068	MCI	30/30	NA	NA
RA 082	MCI	29/30	Trails B 203	9/13.
AK 084	MCI	30/30	85/145	12/13.
NE 001	Control	29/30	NA	NA
DB 002	Control	30/30	NA	NA
GB 006	Control	28/30	NA	NA
GA 009	Control	29/30	NA	NA
HS 014	Control	30/30	NA	NA
LH 017	Control	29/30	NA	NA
RM 018	Control	29/30	NA	NA
HM 019	Control	30/30	NA	NA
EH 027	Control	30/30	66/94	15/14
RB 034	Control	28/30	NA	NA
NZ 042	Control	27/30	NA	NA
EC 037	Control	30/30	NA	NA
AW 041	Control	30/30	NA	NA
PK 047	Control	30/30	NA	NA
RC 048	Control	30/30	NA	NA
EZ 055	Control	29/30	NA	NA

NL	061	Control	30/30	60/90	12/13.
HM	053	Control	30/30	NA	NA
RM	056	Control	28/30	NA	NA
MH	060	Control	29/30	NA	NA
MB	058	Control	29/30	nA	NA
JG	070	Control	30/30	NA	NA
IT	072	Control	28/30	78/124	12/13.
LT	076	Control	29/30	75/154	12/12.
RT	077	Control	30/30	59/147	11/11.
SB	078	Control	28/30	NA	NA
FG	085	Control	28/30	Trails A--113	Clox 1--15
DH	086	Control	30/30	118/unable	6/5.
CW	088	Control	26/30	NA	NA

© 2020 by the author. Licensee MDPI, Basel, Switzerland. This article is an open access article distributed under the terms and conditions of the Creative Commons Attribution (CC BY) license (http://creativecommons.org/licenses/by/4.0/).
